# WaveGFD: Data and methods for numerically solving the wave equation using a meshless Generalized Finite Differences Scheme

**DOI:** 10.1016/j.dib.2024.110776

**Published:** 2024-07-31

**Authors:** Gerardo Tinoco-Guerrero, Francisco J. Domínguez-Mota, José A. Guzmán-Torres, José G. Tinoco-Ruiz

**Affiliations:** a Civil Engineering Faculty, Universidad Michoacana de San Nicolás de Hidalgo, Morelia, Michoacán, 58020, Mexico; b Faculty of Physical-Mathematical Sciences, Universidad Michoacana de San Nicolás de Hidalgo, Morelia, Michoacán, 58020, Mexico

**Keywords:** Meshless method, Generalized finite difference, Wave equation, Irregular regions, Numerical solution

## Abstract

WaveGFD is a repository inspired by the development and analysis of meshless finite difference schemes for the wave equation in highly irregular domains, such as polygonal approximations of geographical regions.

These methods' innovative approach allows to address the complexity of solving partial differential equations in highly irregular regions. They stand out for their precision and efficiency. The proposed methodology overcomes the limitations of conventional techniques, promising applicability in a broad spectrum of complex physical problems.

The repository includes the methods necessary to find approximations to the solution of the wave equation, a set of test data consisting of clouds of points whose complexity varies from the unitary square (for comparison purposes) to regions that approximate actual geographic areas, examples of use, and results obtained with the proposed data and methods.

WaveGFD considers the importance of having methods suitable for efficient implementations (*i.e.*, that can be executed rapidly without the need for extensive and powerful computers) that can obtain satisfactory results when solving problems that may arise in different areas of engineering, such as civil and structural engineering (analysis of the vibrations in buildings and bridges), aerospace engineering (studies of the aeroelasticity of aircraft wings), electrical and telecommunications engineering (models of the propagation of electromagnetic waves), among others.

Specifications Table

**Table:** 

Subject	Computational Mechanics, Applied Mathematics, Computational Mathematics, Mathematical Physics, Numerical Analysis.
Specific subject area	Geometric approximations to geographic regions to apply a meshless approach to the generalized finite difference method, including approximation methods.
Type of data	Graphs, Figures, CSV files. Analysed, Filtered, Processed.
Data collection	The authors generated all the data for this dataset. To obtain the irregular clouds of points that are geometrical approximations to real geographical locations, the selection of the study regions was made considering the demands of the academic community as well as the interests of the authors, thus selecting the regions CUI (Cuitzeo Lake, Mexico), DOW (Pátzcuaro Lake, Mexico. Southern zone), MIC (State of Michoacán, Mexico), PAT (Pátzcuaro Lake, Mexico), and ZIR (Zirahuén Lake, Mexico) due to local interest of the authors, and the regions BAN (Banderas Bay), BLUE (Blue Lagoon), ENG (United Kingdom), GIB (Strait of Gibraltar), HAB (Havana Bay), TIT (Titicaca Lake), TOB, (Toba Lake), UCH (Uchinskoye Reservoir), and VAL (Valencia Lake) as internationally known examples. In all cases, Google maps was used to obtain satellite images of the regions of interest. Subsequently, the borders of the regions were drawn by using the “Contour Creator” software, within UNAMalla. The boundary coordinates were scaled and normalized to fit in the unit square [0,1]×[0,1]. Subsequently, using the “GFD Cloud Generator” software, freely published on GitHub by the authors, the respective clouds of points were obtained for each one of the regions. The authors designed and implemented the methods in Python, following the ideas presented in the related research article.
Data source location	• Institution: Universidad Michoacana de San Nicolás de Hidalgo. • City: Morelia, Michoacán. • Country: México. • Latitude and longitude (and GPS coordinates, if possible) for collected samples/data: 19°41′21.1″N 101°12′05.8″W
Data accessibility	Repository name: GitHub Data identification number: 10.5281/zenodo.10962627 [8] Direct URL to data: https://github.com/gstinoco/WaveGFD
Related research article	Tinoco-Guerrero G., Arias-Rojas H., Guzmán-Torres J.A., Román-Gutiérrez R., and Tinoco-Ruiz J.G., A meshless finite difference scheme applied to the numerical solution of wave equation in highly irregular space regions. Computers & Mathematics with Applications. 136 (2023) 25–33. https://doi.org/10.1016/j.camwa.2023.01.035

## 1 Value of the Data

• The dataset includes a collection of clouds of points for testing problems and comparing results. The clouds are labeled, showing each interior node and boundary node. Labeling allows the problem's boundary conditions to be correctly adjusted and gives information about the interior nodes where the solution of the corresponding equation should be computed.
• The clouds of points in this dataset can be freely used to test finite difference, finite volume, finite element methods, among other techniques for solving partial differential equations. Their use is not limited to the numerical solution of the wave equation; they can be used as a test set for many different equations and problems that do not require the structure of a structured mesh.
• The methods in the repository allow to compute approximations to the solution of the wave equation in highly irregular regions using clouds of points such as those in the dataset. Furthermore, small changes can be quickly adjusted to solve other partial differential equations that model the behavior of different physical phenomena.
• Other methods in the repository allow one to quickly calculate the mean square error for the computed numerical solutions, appropriately graph different types of problems on the attached data set, and find the neighboring nodes of each node in a cloud of points, which is of utmost importance for the correct use of meshless generalized finite difference schemes.

## 2 Background

Building this repository of data and methods is motivated by two goals: to advance the understanding of how methods for the numerical solution of partial differential equations in irregular regions operate without the need for a mesh structure and to have a set of data that can be used to compare the performance of different methods.

On the one hand, the theoretical basis of generalized finite difference methods allows their application to numerically solve a wide variety of partial differential equations in irregular regions; however, in general, their use has been limited to areas that could be meshed with logically rectangular meshes; The methods in this repository allow their application to numerically solve the wave equation without requiring a given mesh structure. In addition, the methods presented in this repository allow to graph the results obtained by the methods directly and calculate the mean square error for numerical solutions.

In addition, having an established test data set allows direct comparisons with the proposed methods. It also provides a starting point for comparing different types of solvers in the same regions to determine the most effective and precise techniques.

Therefore, this repository is valuable for expanding and applying other methods developed to solve partial differential equations, particularly for hyperbolic equations like those presented in [[1], [2], [3], [4], [5]].

Methodologically, compiling these data and methods involved meticulous documentation in finite difference and finite element schemes, as well as the use of different software techniques to obtain the test data. Various algorithms and methods were implemented to ensure that the data can be applied to other engineering problems involving the solution of partial differential equations.

The final objective of this repository is to provide versatile methods and data for researchers and professionals, aiming to promote collaboration and contribute to generating advances in the field of numerical solutions of partial differential equations through robust and computationally cheap methods.

## 3 Data Description

**Fig. 1:**
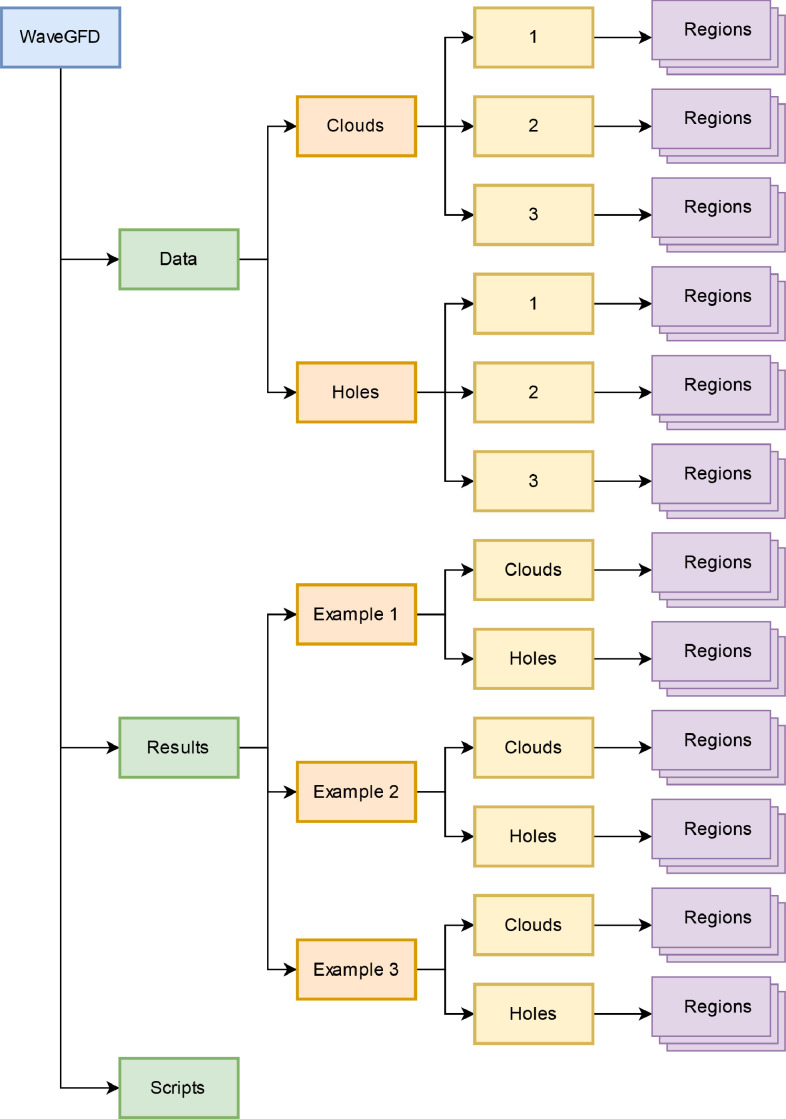
Directory tree of the repository (files excluded).

This repository comprises 609 files, divided into three main directories and subdirectories. The structure of the repository presented is simple and makes it easy to track the information. At the outset, the directory tree has the structure shown in Fig. 1, where the Data, Results, and Methods files are directly separated.

Of the total files, 11 files are files with Python methods, which are in the root directory and the ``Scripts'' directory:

• **/Example_1.py**. This file presents an example of the application of the generalized finite difference method for the wave equation using initial and boundary conditions:

f=cos(πt)sin(π(x+y))

• **/Example_2.py**. This file presents an example of the application of the generalized finite difference method for the wave equation using initial and boundary conditions:

$f=\cos\left(\pi ct\sqrt{2}\right)\sin\left(\pi x\right)\sin\left(\pi y\right)$

• **/Example_3.py.** This file presents an example of applying the generalized finite difference method for the wave equation, simulating a water drop as an initial and reflective boundary condition.
• **/Wave_2D.py.** Main repository file. It contains the methods necessary to compute a numerical solution to the wave equation.
• **/Scripts/Errors.py.** Presents the methods necessary to compute the mean square error of the calculated numerical solutions.
• **/Scripts/Gammas.py.** It contains the routine necessary to calculate the Gamma values for the generalized finite difference method.
• **/Scripts/Graph.py.** This file contains the methods for generating solution graphs and, where appropriate, saving them as images or videos in .mp4 format.
• **/Scripts/Neighbors.py.** It allows searching for the neighbors of each node in a cloud of points, which will be used by the generalized finite difference method.

**Fig. 2:**
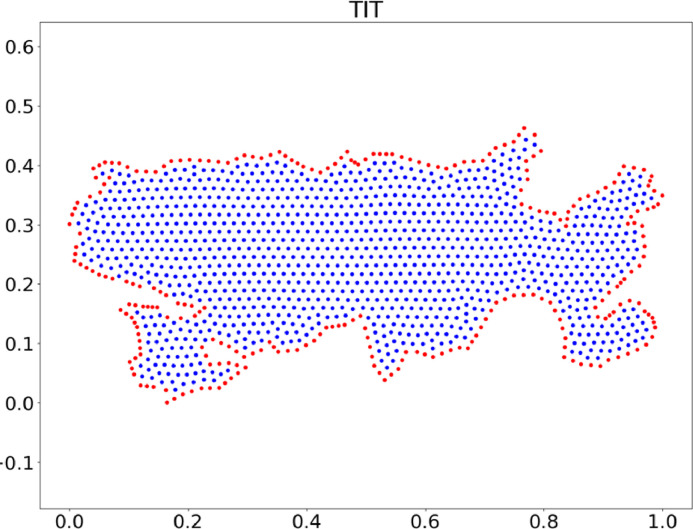
Example of a Cloud of Points. Titicaca lake.

The ``Data'' directory contains two subfolders, ``Clouds'' and ``Holes.'' Each subfolder contains three folders, each with 16 clouds of points for different regions. Each region consists of four files: two figures (in EPS and PNG format) that graphically show the distribution of the nodes in each cloud of points and their labeling to determine the border nodes and the interior nodes, as shown in Fig. 2; and the region's data (in CVS format); this data is stored in two files: *_***p.csv*** and *_***tt.csv***. These files contain the coordinates of the nodes and a triangulation correlation, respectively. Table 1, Table 2 show an example with some of the data from the ``TIT'' cloud in folder 2.

**Table 1:** Structure of TIT_p.csv file.

x-coordinates	y-coordinates	Boundary flag
0.0153141846	0.0192003609	1
0.0377035349	0.0096001804	1
0.0600928853	0	1
0.0874146317	0.0060273985	1
…	…	…
0.1891414493	0.0758787178	0
0.2579077708	0.07382601	0
0.2932067794	0.0827686344	0
0.2270873129	0.0893064604	0

**Table 2:** Structure of the TIT_tt.csv file.

Node 1	Node 2	Node 3
393	95	96
99	391	98
95	93	94
91	95	393
…	…	…

**Fig. 3:**
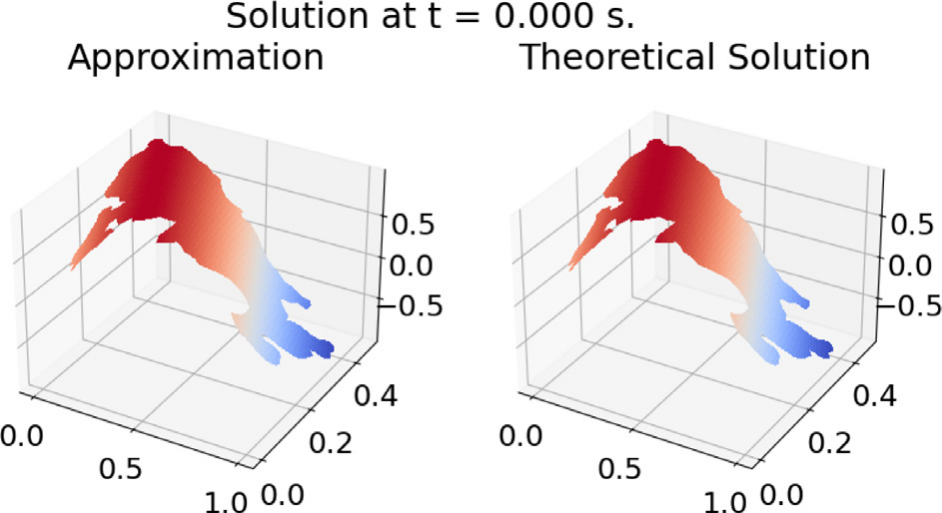
Results of Example 1 on TIT_3. *t* = 0.0 s.

**Fig. 4:**
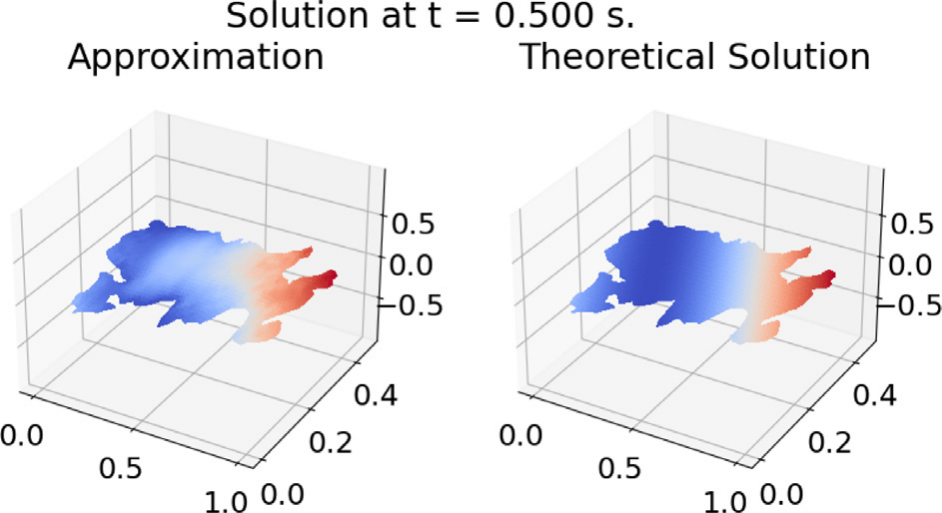
Results of Example 1 on TIT_3. *t* = 0.5 s.

**Fig. 5:**
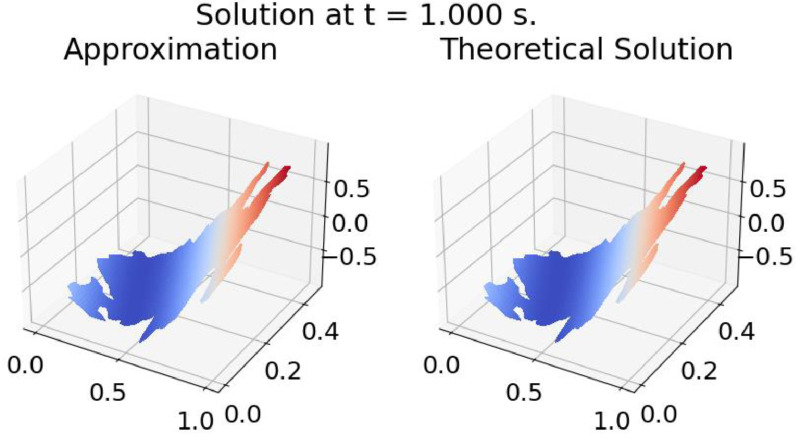
Results of Example 1 on TIT_3. *t* = 1.0 s.

The ``Results'' directory contains three directories: “Example 1”, ``Example 2”, and ``Example 3”. Each of these directories contains two subdirectories, ``Clouds'' and ``Holes,'' which, in turn, contain one directory for each of the test regions (BAN, BLU, CAB, CUA, CUI, DOW, ENG, GIB, HAB, MIC, PAT, TIT, TOB, UCH, VAL, ZIR). Within each test region directory, it is possible to find nine files with PNG graphics showing the results of the corresponding test at three different time levels (*t* = 0 s, 0.5 s, 1 s), as in Fig. 3, Fig. 4, Fig. 5; in these results, the value of the solution for each problem is shown on the z-axis. In addition, there are three videos in mp4 format containing test results at different time levels.

## 4 Experimental Design, Materials and Methods

In this work and within the repository, there are two types of data and information: the data of the clouds of points as test regions for meshless methods and a generalized finite difference method for numerically solving the wave equation in irregular regions using a meshless approach; it is essential to describe how both were obtained.

### 4.1 Clouds of points for different space regions

The process for creating the clouds of points in the repository was the same for all regions except for the CAB and CUA regions, which are not geometric approximations of real geographic locations and can be used to compare the results with mesh-based methods.

To exemplify the process, how the point clouds were obtained for the TIT region (Lake Titicaca) is shown.

1. The geometry of the geographic region will be based on the geometries found on maps for public use. In this case, an image of the region of interest is taken from Google Earth (Fig. 6).

**Fig. 6:**
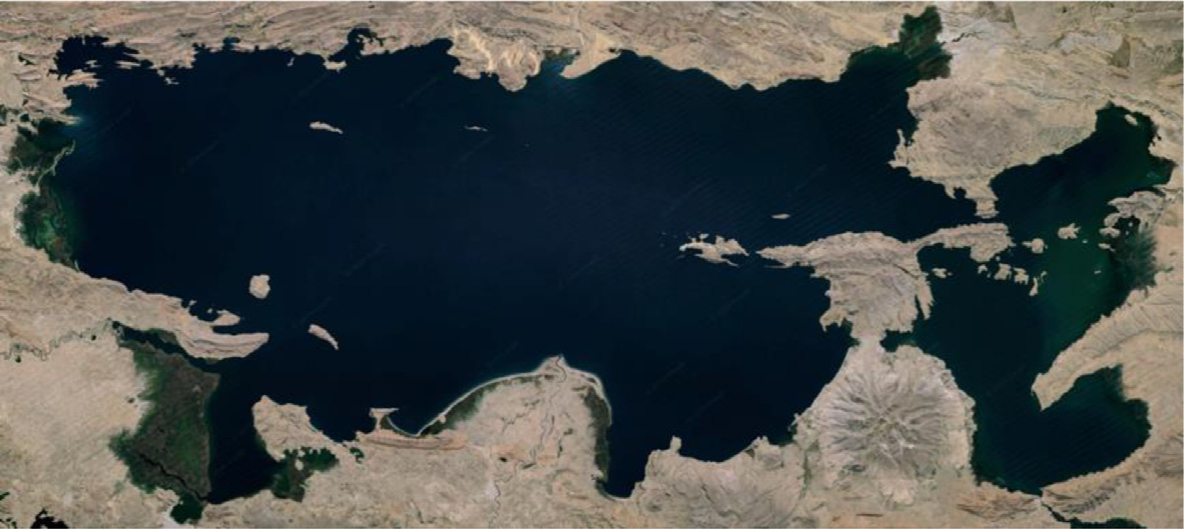
Titicaca Lake. Retrieved from [6].
2. Once one has an image of the region, it can be imported into the "Contour Creator" Software included with "UNAMalla" (Fig. 7).

**Fig. 7:**
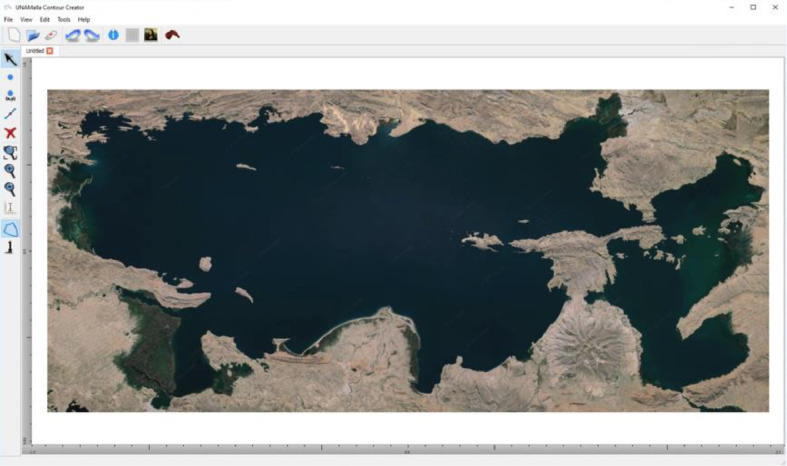
Contour Creator from UNAMalla.
3. Within Contour Creator, drawing the region's border by placing different points is possible. These points can be placed in as much detail as desired (Fig. 8).

**Fig. 8:**
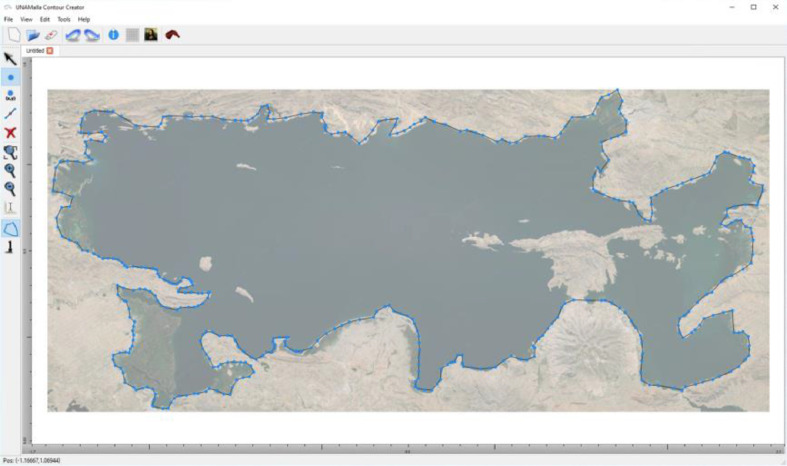
Boundary selection on Contour Creator.
4. As an optional step, a mesh that smoothens the boundaries can be generated directly in UNAMalla.
5. The file containing the region border can now be processed using the "Cloud Generation" package, which the authors published for free on GitHub.

### 4.2 Cloud generation package

The ``Cloud Generation'' package is a free-access package on GitHub (https://github.com/gstinoco/Cloud-Generation), published by the authors of this work. It allows the generation of clouds of points, with uniform and non-uniform distributions, from a region's boundary or mesh.

In all cases presented in this repository, the border of the region obtained from Contour Creator was used directly to create the point clouds. The process is as follows.

1. The region's boundary is imported from a ``.con'' file (from Contour Creator), a ``.mat'' file (from MATLAB), or a “.csv” file.
2. The regions were scaled to enter the unit square [0,1]×[0,1], using the following formula:
  $p_{ix}=\frac{p_{ix}-\min\left(x\right)}{\max(x,y)-\min(x,y)},\;p_{iy}=\frac{p_{iy}-\min\left(y\right)}{\max(x,y)-\min(x,y)}$ Where $p_{ix}$ and $p_{iy}$ represent the coordinates of each node in x and y, respectively; min(x) and min(y) are the maximum value of in the x and y directions, respectively; max(x,y)=max(max(x),max(y)), and min(x,y)=min(min(x),min(y)).
3. Once the regions have been scaled, the maximum distance between two consecutive nodes is calculated; this distance will be considered the average distance between the region's nodes.
4. With the border nodes, a dmsh polygon is generated; This polygon will serve as the input to create a dmsh triangulation [7].
5. Once the region's nodes are obtained, it is essential to correctly label each one, differentiating between boundary and interior nodes. To do this, a buffered polygon is created with the original nodes, and it is checked which of the triangulation nodes are on this polygon or in its buffer. All the nodes that meet the previous condition will be labeled as boundary nodes (Fig. 9).

**Fig. 9:**
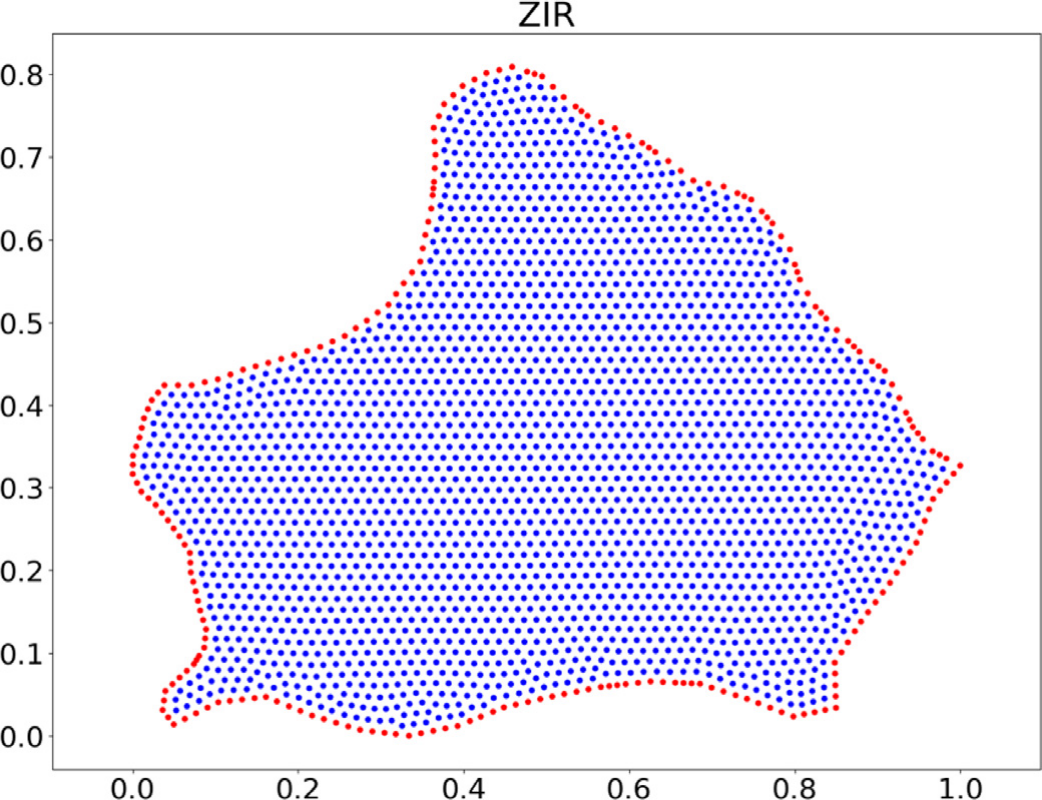
Uniform cloud of points for ZIR region.
6. These nodes can be used as a first cloud of points with a uniform distribution; However, node randomization can also be performed to avoid ``regular'' clouds.
7. In randomization, each node moves randomly between 0 % and 5 % of its original position to create clouds with a non-uniform distribution of nodes (Fig. 10).

**Fig. 10:**
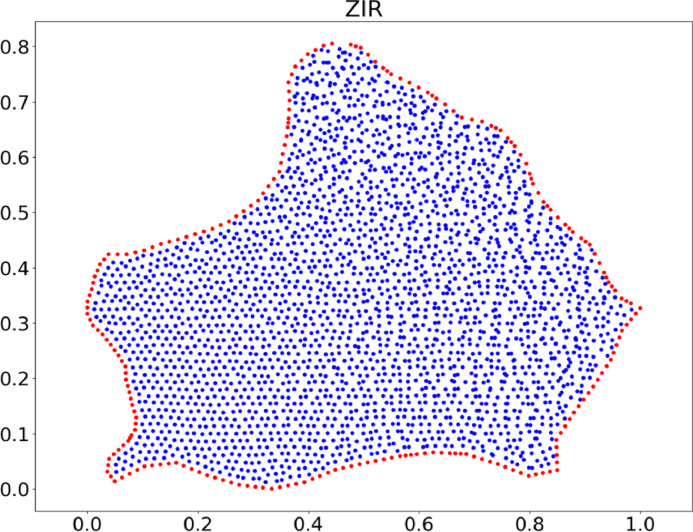
Non-uniform cloud of points for ZIR region.
8. Non-simply connected clouds can also be generated and randomized. When generating the dmsh triangulation, it is possible to “subtract” the region where a “hole” is present inside the boundary. From here, the process is the same as before. Examples of this procedure are depicted in Fig. 11, Fig. 12.

**Fig. 11:**
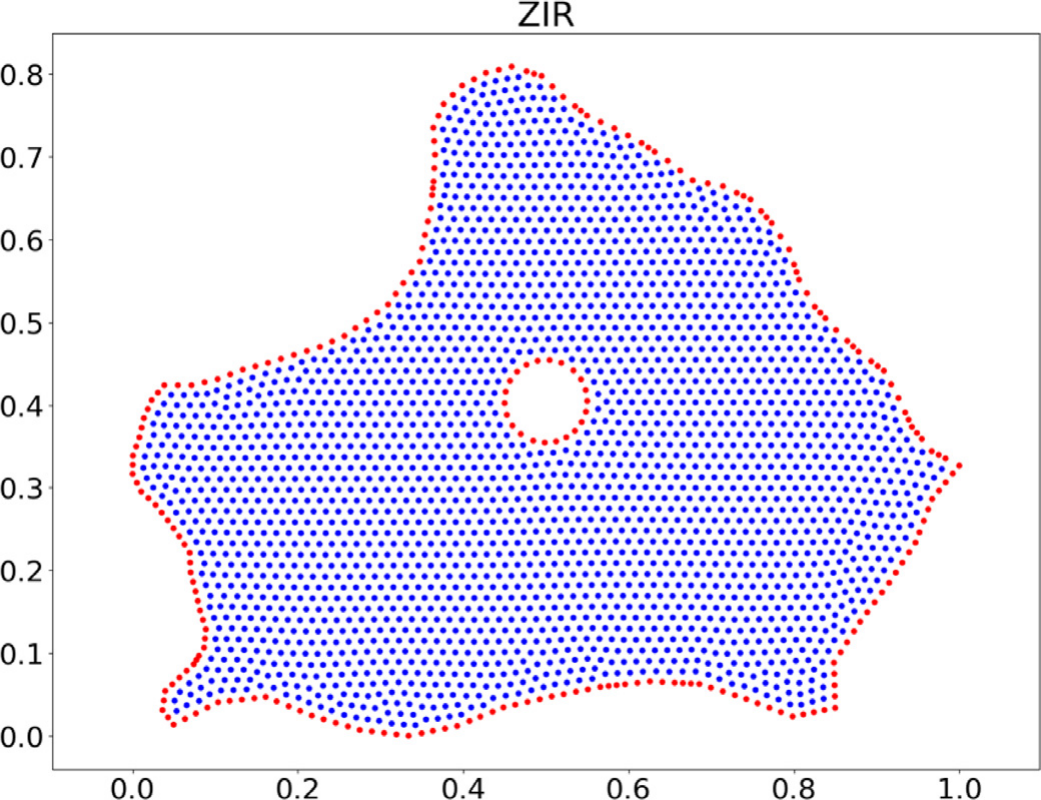
Uniform cloud of points for ZIR region with a hole.

**Fig. 12:**
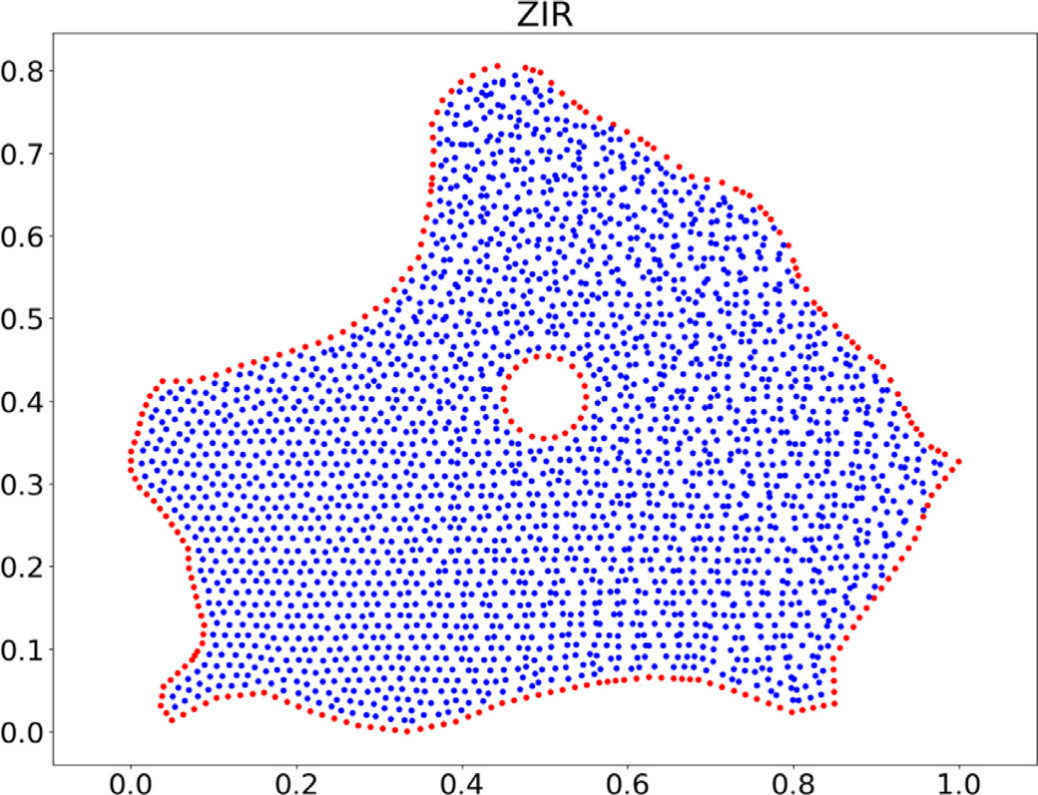
Non-uniform cloud of points for ZIR region with a hole.

## 5 A meshless approach for generalized finite difference methods

The presented meshless approach for generalized finite difference methods arises from the idea of computing a numerical solution for the wave equation problem:

(1) $$\frac{\partial^{2}u}{\partial t^{2}}=c^{2}\left(\frac{\partial^{2}u}{\partial x^{2}}+\frac{\partial^{2}u}{\partial y^{2}}\right),\;\Omega \times \left[0,T\right],\;c\in \;R,$$

subject to the conditions

$\begin{array}{c} u(x,y,0)=h(x,y,0),\;\;\;\;\;\;(x,y)\in \Omega \\ u\left(x,y,t\right){|}_{\partial \Omega }=h\left(x,y,t\right),\;\;\;\left(x,y\right)\in \Omega ,\;t\in \left[0,T\right]\\ \overset{˙}{u}\left(x,y,t\right){|}_{t=0}=g\left(x,y\right),\;\;\;\;\left(x,y\right)\in \Omega , \end{array}$

where Ω is the region of interest.

Since a second temporal derivative is introduced in the equation, a centered scheme can be used for temporal discretization; for an arbitrary node $p_{0}=\left(x_{0},\;y_{0}\right)$; this discretization would be of the form:

(2) $$u_{p_{0}}^{k+1}=2u_{p_{0}}^{k}-u_{p_{0}}^{k-1}+{\left(c\Delta t\right)}^{2}{\left(\frac{\partial^{2}u}{\partial x^{2}}+\frac{\partial^{2}u}{\partial y^{2}}\right)}_{p_{0}}$$

It is important to note that, in this scheme, two time steps are necessary; then, following the idea presented in [1], the second time step (which would require step 0 and step −1) can be approximated as:

(3) $$u_{p_{0}}^{1}=u_{p_{0}}^{0}+\Delta tg\left(x,y\right)+\frac{{\left(c\Delta t\right)}^{2}}{2}{\left(\frac{\partial^{2}u}{\partial x^{2}}+\frac{\partial^{2}u}{\partial y^{2}}\right)}_{p_{0},\;t=0}$$

For the spatial discretization, considering the second-order linear operator,

(4) $$Lu\;=\;Au_{xx}+Bu_{xy}+Cu_{yy}+Du_{x}+Eu_{y}+F$$

where A, B, C, D, E, and F are given functions. For an arbitrary cloud of points, the value of Lu an arbitrary node $p_{0}=\left(x_{0},\;y_{0}\right)$ can be approximated using values of u at some neighbor nodes $p_{i}=\left(x_{i},\;y_{i}\right)$, i=1,2,…,q. Thus, a finite-difference scheme can be applied in p_0 as a linear combination

(5) $$L_{0}u=\Gamma_{0}\;u\left(p_{0}\right)+\Gamma_{1}u\left(p_{1}\right)+\ldots +\Gamma_{q}u\left(p_{q}\right)=\sum_{i=0}^{q}\Gamma_{i}u\left(p_{i}\right),$$

where $\Gamma_{0},\Gamma_{1},\ldots ,\Gamma_{q}$ are suitable weights.

It is said that a finite difference scheme $L_{0}$ is consistent with the linear operator L if the local truncation error τ satisfies

(6) $$\tau =\;{\left[Lu\right]}_{p_{0}}-{\left[L_{0}u\right]}_{p_{0}}.$$

This consistency condition yields the linear system

(7) $$\left(\begin{array}{ccc} \Delta x_{1} & \ldots & \Delta x_{q}\\ \Delta y_{1} & \ldots & \Delta y_{q}\\ \begin{array}{c} {\left(\Delta x_{1}\right)}^{2}\\ \Delta x_{1}\Delta y_{1}\\ {\left(\Delta y_{1}\right)}^{2} \end{array} & \begin{array}{c} \ldots \\ \ldots \\ \ldots \end{array} & \begin{array}{c} {\left(\Delta x_{q}\right)}^{2}\\ \Delta x_{q}\Delta y_{q}\\ {\left(\Delta y_{q}\right)}^{2} \end{array} \end{array}\right)\left(\begin{array}{c} \Gamma_{1}\\ \Gamma_{2}\\ \begin{array}{c} \Gamma_{3}\\ \vdots \\ \Gamma_{q} \end{array} \end{array}\right)=\left(\begin{array}{c} D\left(p_{0}\right)\\ E\left(p_{0}\right)\\ \begin{array}{c} 2A\left(p_{0}\right)\\ B\left(p_{0}\right)\\ 2C\left(p_{0}\right) \end{array} \end{array}\right)$$

Applying this linear for the linear operator

(8) $$Lu\;=\;{\left(c\Delta t\right)}^{2}\left(\frac{\partial^{2}u}{\partial x^{2}}+\frac{\partial^{2}u}{\partial y^{2}}\right)$$

The obtained $\Gamma_{i}$ values, the define the generalized finite difference schemes:

(9) $$u_{p_{0}}^{1}=u_{p_{0}}^{0}+\Delta tg\left(x,y\right)+\frac{1}{2}\sum_{i=0}^{q}\Gamma_{i}u_{i,p_{0}}^{0}$$

(10) $$u_{p_{0}}^{k+1}=2u_{p_{0}}^{k}-u_{p_{0}}^{k-1}+\sum_{i=0}^{q}\Gamma_{i}u_{i,p_{0}}^{k}$$

In the repository, these schemes are applied to all the generated clouds of points for three different examples:

1. Example 1:
  • f(x,y,t)=cos(πt)sin(π(x+y)) as initial and boundary conditions.
  • g(x,y)=0
  • T=[0,1]
  • Δt=0.0005
2. Example 2:
  • $f\left(x,y,t\right)=\cos\left(\pi ct\sqrt{2}\right)\sin\left(\pi x\right)\sin\left(\pi y\right)\;$as initial and boundary conditions.
  • g(x,y)=0
  • T=[0,1]
  • Δt=0.0005
3. Example 3:
  • $f\left(x,y,t\right)=0.2e^{-2000({\left(x\;-\;r_{x}-\;ct\right)}^{2}+\;{\left(y\;-\;r_{y}-\;ct\right)}^{2})}$ as the initial condition.
  • f(x,y,t)=0 as the boundary condition.
  • g(x,y)=0
  • T=[0,1]
  • Δt=0.0005

## Limitations

Now, the dataset is limited to the selected regions. Nonetheless, the number of regions will increase over time. Following the process described in this work, anyone can use the methods in the repositories to create their dataset according to their interests.

The complete data from the numerical results for the generalized finite difference method could not be saved in the repository due to file-size limitations; nevertheless, executing the examples allows the results to be computed and saved.

The current methods on the repository can deal only with Dirichlet boundary conditions, which can limit the application to many real-life scenarios. Nevertheless, the dataset is currently prepared to deal with Neumann and Robin boundary conditions that will be addressed in the future by the methods.

## Ethics Statement

The authors of this dataset meet the ethical requirements for publication in Data in Brief and do not involve human subjects, animal experiments, or any data collected from social media platforms.

## CRediT authorship contribution statement

**Gerardo Tinoco-Guerrero:** Conceptualization, Methodology, Writing – original draft, Software, Formal analysis, Investigation. **Francisco J. Domínguez-Mota:** Supervision, Project administration, Funding acquisition. **José A. Guzmán-Torres:** Methodology, Validation, Investigation. **José G. Tinoco-Ruiz:** Conceptualization, Methodology, Supervision, Formal analysis.

## Data Availability

WaveGFD (Original data) (GitHub)
